# Overall Survival and Response to Systemic Therapy in Metastatic Extrauterine Leiomyosarcoma

**DOI:** 10.1155/2016/3547497

**Published:** 2016-05-29

**Authors:** A. N. Shoushtari, J. Landa, D. Kuk, A. Sanchez, B. Lala, N. Schmidt, C. Okoli, P. Chi, M. A. Dickson, M. M. Gounder, M. L. Keohan, A. M. Crago, W. D. Tap, S. P. D'Angelo

**Affiliations:** ^1^Sarcoma Oncology Service, Department of Medicine, Memorial Sloan Kettering Cancer Center, 1275 York Avenue, New York, NY 10065, USA; ^2^Weill Cornell Medical College, 1300 York Avenue, New York, NY 10065, USA; ^3^Department of Radiology, Memorial Sloan Kettering Cancer Center, 1275 York Avenue, New York, NY 10065, USA; ^4^Department of Epidemiology and Biostatistics, Memorial Sloan Kettering Cancer Center, 1275 York Avenue, New York, NY 10065, USA; ^5^Department of Surgery, Memorial Sloan Kettering Cancer Center, 1275 York Avenue, New York, NY 10065, USA

## Abstract

*Background*. Leiomyosarcomas (LMS) represent a heterogeneous subset of soft tissue sarcomas. Factors influencing prognosis for patients with metastatic extrauterine LMS (euLMS) are not well described. Limited data are available regarding responses to systemic therapy.* Methods*. We collected clinical and pathologic information for all patients with metastatic euLMS seen at Memorial Sloan Kettering Cancer Center between 1989 and 2012. Objective responses to first-line therapy were analyzed for a subset of patients with available baseline and on-treatment imaging using RECIST 1.1.* Results*. 215 patients with metastatic euLMS had a median overall survival (OS) of 2.6 years from the time of metastasis. Older age, male sex, and ≥3 initial sites of metastasis were associated with worse OS on multivariate analysis. Objective response rate (ORR) in *N* = 113 was 19% overall and 25%, 26%, and 25% for gemcitabine, gemcitabine plus docetaxel, and anthracycline-alkylator combinations. Patients whose tumors objectively responded to first-line therapy had a lower risk of death versus those who did not (Hazard Ratio 0.46; 95% CI: 0.26–0.79, *p* = 0.005).* Conclusions*. Anthracycline- and gemcitabine-based regimens have similar activity in this cohort of euLMS. Prognostic factors for OS include older age, male sex, and ≥3 initial sites.

## 1. Introduction

Leiomyosarcomas (LMS) are a heterogeneous group of smooth muscle neoplasms that can arise in the uterus, extremity, or other primary sites [1]. Together, LMS comprise the most common single soft tissue sarcoma histology (24% of all soft tissue sarcomas), with an incidence of approximately 1.2 cases per 100,000 person-years [2]. Increasing clinical and genetic data suggest LMS arising from an extrauterine primary site represents a distinct subset of tumors [3–5]. Until the identification of KIT positive tumors, many prior analyses of extrauterine LMS (euLMS) were likely contaminated by unrecognized gastrointestinal stromal tumors, limiting our understanding of this disease entity. A recent single-center, retrospective review of 332 patients with primary euLMS identified size and grade as distinct factors that influenced disease-specific survival [1]. Little is known, however, about factors influencing prognosis for patients with metastatic euLMS. A series of all LMS subtypes included 101 with euLMS and found that the sole factor influencing OS was a shorter interval from diagnosis to metastatic disease [6].

The choice of systemic therapy agents is extrapolated using prospective and retrospective analyses of LMS pooled from uterine and euLMS, most often utilizing gemcitabine with or without docetaxel or doxorubicin with or without an alkylator such as ifosfamide (AIM) [7–9]. Recently, the results of GeDDiS, a randomized Phase 3 trial comparing gemcitabine plus docetaxel with doxorubicin in the front-line setting, were reported in multiple soft tissue sarcoma subtypes, including uterine and euLMS [10]. The primary endpoint of 24-week PFS was identical between arms, 46% each. Two separate, randomized Phase 3 trials identified agents in the second-line setting that provided clinical benefit for patients with any type of LMS. Trabectedin was recently FDA-approved for treatment of LMS previously treated with an anthracycline on the basis of a significant PFS benefit versus dacarbazine (4.3 versus 1.5 months, *p* < 0.001) [11]. Eribulin significantly prolonged OS versus dacarbazine in a cohort of patients with LMS or liposarcoma, although an exploratory subgroup analysis suggested that the majority of benefit was limited to patients with liposarcoma [12]. A prospective study of 2nd-line therapy with gemcitabine or gemcitabine plus docetaxel in euLMS reported a response rate of 14% and 5%, respectively [13].

Response rates to first-line systemic therapy in advanced euLMS have been reported in small retrospective case series utilizing anthracycline- or alkylator-based regimens such as AIM. One series that reported ORR specifically in euLMS included 32 patients who either received doxorubicin or other single-agent alkylators (*n* = 19), ifosfamide (*n* = 9), or AIM (*n* = 4); overall, the pooled ORR was 22% [14]. Another series of 18 patients, the majority of which received doxorubicin (*n* = 9) or epirubicin plus ifosfamide (*n* = 6), reported a response rate of 33% [15].

In euLMS, it is not well described whether the choice of systemic therapy agent is associated with differences in response rate or whether the objective response to first-line systemic therapy influences clinical outcomes. We therefore undertook a retrospective, single-center analysis of patients with advanced euLMS to analyze the association of clinicopathologic factors, first-line systemic therapy agent, and objective response to first-line systemic therapy with survival from the time of metastatic disease.

## 2. Methods

After institutional IRB approval, patients were identified from a database query who met the following criteria: (1) ≥1 visit at Memorial Sloan Kettering Cancer Center (MSKCC) between 1989 and 2012; (2) a pathologic diagnosis of leiomyosarcoma arising from an extrauterine primary site; and (3) developed metastatic disease at any point from diagnosis to last follow-up visit. Clinicopathologic data included age at first diagnosis, status at presentation (primary versus metastatic), year of development of metastatic disease, time interval between primary and metastatic diseases, sex, depth, grade (utilizing the Fédération Nationale des Centres de Lutte Contre le Cancer (FNCLCC) system), primary tumor size, site, Karnofsky Performance Status (KPS) at time of metastasis, margins, number of metastatic sites at first presentation of advanced disease, presence versus absence of lung or liver metastases, treatment type at first recurrence, and first-line systemic therapy agent. Patients with missing clinicopathologic data were included and data were marked as “unknown” for that specific category.

Treatment type at first recurrence was analyzed only for patients who initially presented with localized disease and characterized as systemic therapy only, local therapy only (surgery, embolization), or combination systemic and local therapy. Systemic therapy agents in this retrospective cohort were up to the discretion of the treating physician and were classified for our analysis as follows: doxorubicin alone, liposomal doxorubicin alone, gemcitabine alone, gemcitabine plus docetaxel, anthracycline-alkylator combinations (doxorubicin, ifosfamide, and mesna [AIM], AIM plus dacarbazine, and doxorubicin plus dacarbazine), tyrosine kinase inhibitors (sunitinib, sorafenib), and other combination or single agents. Patients were considered evaluable for response to first-line therapy if at least 1 CT or MRI was available both before and at least three weeks after initiating first-line therapy. Radiographic responses to first-line systemic therapy were assessed by a radiologist blinded to treatment (JL) using Response Evaluation Criteria in Solid Tumors (RECIST) 1.1, with the exception that repeat assessments to confirm response were not required in this retrospective analysis. Time to treatment failure (TTF) was calculated as start of systemic therapy until date of progressive disease by RECIST 1.1 or new therapy, whichever was first.

Patient demographics are presented by median and range for continuous variables and by frequency and percentage for categorical variables. Overall survival (OS) was calculated from date of development of metastatic disease to date of death or last follow-up. Patients alive at last follow-up were censored. The Kaplan-Meier method, log-rank test, and Cox proportional hazards regression were used for estimation, testing, and regression modeling of overall survival, respectively. For patients with RECIST response data, the relationship between response and OS was evaluated. OS in this subset of patients was calculated from date of start of chemotherapy to date of death or last follow-up. RECIST response was treated as a time dependent covariate in the Cox proportional hazards model. For chemotherapy regimens, time to treatment failure (TTF) is calculated as date from start of chemotherapy to date of progression by RECIST 1.1 or first day of subsequent-line chemotherapy. The Kruskal-Wallis test was used to compare TTF between the different chemotherapy regimens. *p* values < 0.05 are considered significant. All analysis was done using R version 3.1.1 (https://cran.r-project.org/).

## 3. Results

### 3.1. Overall Survival

A total of 215 patients met inclusion criteria. See Table 1 for demographics. Median age was 56 (range: 24–85), and 63% of patients were female. A majority of patients initially presented with localized disease (61%). The median primary tumor size was 9 cm (range: 2–30 cm), and the most common primary site was in the abdomen or retroperitoneum (*n* = 151, 70%). The majority of primary tumors were high grade (*n* = 176, 82%) and deep (*n* = 196, 91%). For patients diagnosed with localized disease, the median time to developing metastases was 15.8 months (range: 2.1–232 months). The median number of metastatic sites was 2 (range: 1–8). The most common sites of metastatic disease were lung (53%) and liver (26%). KPS at time of metastasis was relatively preserved in this cohort; only 10 of 215 (5%) had KPS of 70 or below (ECOG ≥ 2), and 72 (33%) had an unknown performance status.

**Figure 1:**
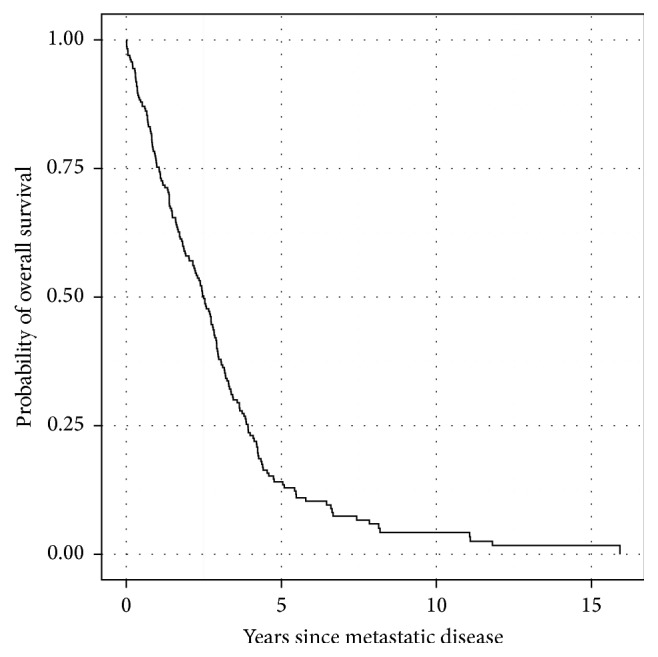
Overall survival from date of metastatic disease. Median OS was 2.6 years.

Median follow-up amongst survivors is 14.6 years. There were 190 deaths. Median OS from time of diagnosis of metastatic disease is 2.6 years (Figure 1). On univariate analysis, older age, male sex, shorter interval between primary and metastatic disease, and higher number of initial metastatic sites were significantly associated with an increased risk of death (Table 2). Median OS for patients above versus below the median age of 56 was 2.4 versus 2.8 years (*p* = 0.006); 2.9 versus 2.2 years for females versus males (*p* = 0.005); and 2.0 versus 2.8 years for ≥3 metastatic sites versus 1-2 metastatic sites (*p* = 0.002). On multivariate analysis (Table 3), (Hazard ratio [HR] and 95% confidence interval): older age (HR = 1.36; 1.01–1.84), male sex (HR = 1.42; 1.05–1.92), and higher number of initial metastatic sites (HR = 1.49; 1.10–2.03) remained significant. The time to development of metastatic disease was no longer significant (HR = 0.92; 0.84–1.01). The other variables assessed were not associated with OS from time of development of metastatic disease on univariate analysis.

### 3.2. First-Line Systemic Therapy

**Figure 2:**
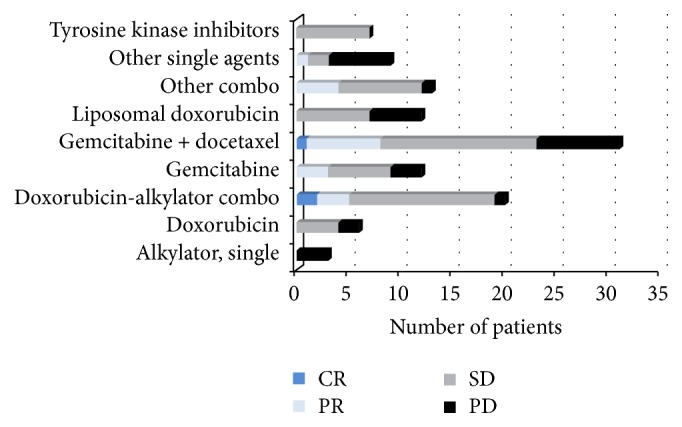
Response to first-line systemic therapy regimens. Objective responses were seen with gemcitabine + docetaxel (8/31, 26%), gemcitabine (3/12, 25%), and doxorubicin-alkylator combinations (5/20, 25%). CR = complete response, PR = partial response, SD = stable disease, and PD = progressive disease.

One hundred thirteen patients received first-line systemic therapy agents and were evaluable for objective response. The first-line chemotherapy classes and objective responses are depicted in Figure 2. The objective response rate (ORR) to first-line chemotherapy was 19% overall (21 of 113). The majority of regimens included either gemcitabine (*n* = 47, 42%) or anthracycline (*n* = 42, 37%). The most common regimens were combined gemcitabine plus docetaxel (*n* = 31, 27%) and combined anthracycline plus alkylator (*n* = 20, 18%). The ORR did not vary significantly between these two groups (26% versus 25%, *p* = 1.0). Objective responses were seen in both combination regimens and with single-agent gemcitabine but not single-agent doxorubicin or liposomal doxorubicin. For a detailed list of all regimens, see Supplemental Table 1 of the Supplementary Material available online at http://dx.doi.org/10.1155/2016/3547497.

When comparing classes of first-line therapy, there were no significant differences in TTF (Table 4). For example, the median TTF for doxorubicin alone, anthracycline plus alkylator combinations, and liposomal doxorubicin was 3.3, 3.1, and 2.9 months, respectively (*p* = 0.99). Gemcitabine plus docetaxel versus gemcitabine alone had a median TTF of 5.2 and 4.3 months, respectively (*p* = 0.83). The difference between gemcitabine plus docetaxel and anthracycline plus alkylator combinations was not statistically significant (*p* = 0.95). The small group of patients receiving TKIs (sorafenib, *n* = 6, sunitinib, *n* = 1) had a median TTF of 9.3 months.

To determine the possible effect of prognostic confounders on patients evaluable for response to first-line systemic therapy, the variables significantly associated with OS on univariate analysis (age, sex, metastasis-free interval, and number of initial metastatic sites) were analyzed by therapy class. Only metastasis-free interval (*p* = 0.04) varied significantly across tumor types (Table 5). The metastasis-free interval was shortest for those treated with anthracycline plus alkylator combinations or gemcitabine plus docetaxel (median 0 and 0.23 years) and longest for the *N* = 18 patients who were treated without systemic therapy (1.5 years). Objective responses to first-line systemic therapy were significantly associated with OS. Patients whose tumors have an objective response had a lower risk of death during follow-up than those who had no objective response (HR: 0.46; 95% CI: 0.26–0.79, *p* = 0.005). There was no significant association of systemic therapy type with OS (*p* = 0.40, data not shown). Of the 113 patients evaluable for response, 10 (9%) received trabectedin after first-line therapy (range: 2nd–9th therapies), and no patients received subsequent eribulin. Overall, 98 of 113 patients received at least 1 subsequent systemic therapy.

## 4. Discussion

There are relatively little data published regarding factors influencing survival in patients with metastatic extrauterine leiomyosarcoma. This is to our knowledge the largest study of the impact of clinical and pathologic variables on clinical outcomes in advanced euLMS. Median OS was 2.6 years from the time of developing metastatic disease. Independent predictors of improved outcome included younger age, female sex, and 2 or fewer metastatic sites at first presentation of advanced disease. This is consistent with the trend reported in one study of improved PFS and OS with female sex and younger age in patients with euLMS receiving systemic therapy [14]. Although another study of 50 patients with euLMS found no significant association between clinical variables and outcome [15], this may be due to a difference in population. Their study included all patients with euLMS, not just those who developed metastatic disease, and they had a preponderance of smaller primary extremity tumors that were in the minority in this cohort. It is notable that in this cohort, performance status was not associated with outcome, given that this is a well-established prognostic variable in a large analysis of soft tissue sarcomas [16]. Our analysis is limited, however, by the small percentage of those with lower performance status (<10%) and a relatively high percentage of patients with missing data at the time of metastasis (33%). Our analysis did not demonstrate any association with liver involvement or grade and OS, in contrast to the larger analysis across all sarcoma subtypes [16]. It is not clear whether this is due to an euLMS-specific difference in clinical behavior or whether larger sample sizes are required to appreciate this association.

Our study is the largest to analyze response to first-line systemic therapy in euLMS. The ORR across all chemotherapy types in this study (19%, *n* = 113) is similar to that reported by the Dutch group (22%, *n* = 32) [14]. Both are slightly lower rates than that reported in the smaller Austrian cohort (33%, *n* = 18) [15]. This euLMS cohort includes a large series of patients receiving non-anthracycline-based regimens, including 47 receiving gemcitabine with or without docetaxel and other agents. The response rates of ~25% and median TTF of 4-5 months are comparable to the efficacy seen in randomized trials in uterine LMS, euLMS, and other sarcomas [8, 10, 17]. In this cohort, there was no significant difference in ORR or TTF between gemcitabine with or without docetaxel and anthracycline plus alkylator combinations such as AIM. Notably, patient age and metastasis-free interval were similar between gemcitabine plus docetaxel and anthracycline plus alkylator combinations.

In the first-line GeDDiS trial [10], within the subgroup of all patients with LMS, which included 47 patients with euLMS and 71 with uterine LMS, there was no detectable difference in PFS (HR: 1.12, 95% CI: 0.75–1.66). There was no evidence for subgroup differences between euLMS and uterine LMS (*p* value for interaction = 0.73), and the euLMS subgroup could not detect a significant difference in PFS when analyzed separately (HR: 1.34, 0.98–1.83). It is also notable that a 25% lower dose of gemcitabine was used in GeDDiS versus the other Phase 2/3 published trials (675 mg/m^2^ versus 900 mg/m^2^) [8, 17]. Given these data and the results of our cohort, we believe that gemcitabine with or without docetaxel remains a reasonable alternative to doxorubicin in euLMS.

The median TTF in our study for anthracycline-based regimens was approximately 3 months. There was no difference between doxorubicin monotherapy and doxorubicin plus an alkylator. This lack of median TTF benefit with the addition of an alkylator is echoed by the report across all LMS subtypes (*n* = 147) by the French Sarcoma Group [6]. The TTF value in our report is comparable to the median PFS of 3.8 months reported by the Dutch group focused on euLMS [14]. While liposomal doxorubicin did not lead to any responses in this cohort, it was associated with a similar median TTF to doxorubicin of approximately 3 months. Taken together, these data suggest that the addition of ifosfamide or dacarbazine to doxorubicin or liposomal doxorubicin monotherapy may not provide additional benefit, outside of a higher objective response rate [18], to justify the historically higher rate of adverse events of combination cytotoxic therapy.

This retrospective analysis, as with many others investigating sarcoma subtypes, is limited by selection bias and relatively by low numbers for certain classes of chemotherapy. Patients with more clinically aggressive disease may have been more likely to receive combination cytotoxic regimens, while patients with indolent progression may have been more likely to receive single agents such as sorafenib. This is supported by the data suggesting that patients who received gemcitabine plus docetaxel or doxorubicin had a shorter metastasis-free interval than those receiving a tyrosine kinase inhibitor. This may explain the particularly high median TTF of over 9 months in the small multityrosine kinase inhibitor group; interestingly, this value was higher than the median PFS of 3.2 months seen in all patients with LMS treated on a recent Phase 2 study [19]. Regardless, this does provide equipoise for utilizing single agents when deemed clinically feasible. For example, liposomal doxorubicin could be considered an alternative to anthracycline-alkylator combinations in cases where disease stability would suffice. This is consistent with the results of a prior Phase 2 study showing similar efficacy of liposomal doxorubicin and doxorubicin across multiple sarcoma subtypes [20].

The two randomized Phase 3 trials demonstrating clinical benefit for patients with LMS treated with trabectedin [11] and eribulin [12] in the second-line setting raise interesting questions for future study that cannot be answered by our cohort. During the era analyzed in this cohort, trabectedin was not widely available in the US. Only 1 patient received trabectedin as a first-line therapy, and only 10 patients (9%) received it at any time subsequently. No patients received eribulin in our analysis. As a result, our analysis neither is significantly confounded by nor can add significant insight into the activity of these agents. They represent promising cytotoxic therapies for patients with LMS. For example, in a recent Phase 2 study of trabectedin combined with doxorubicin, 24 of 61 evaluable patients (39%) with euLMS had an objective response [21], underscoring the promise of this agent for this sarcoma subtype.

A significant finding in this study is the fact that patients whose tumors had objectively responding disease to the first systemic treatment had better OS than those who had stable or primary progressive disease. This underscores the importance of understanding how to best select therapies that may benefit patients with advanced euLMS. Given that chemotherapy class alone was not associated with survival in this analysis, one particular regimen cannot be uniformly viewed as superior to any other across all patients.

Taken together, this study suggests that more research must be done to understand which genetic and epigenetic factors influence the response of euLMS to cytotoxic therapy. One active area of investigation is the role that the tumor and immunologic microenvironments play in objective responses [22]. Lymphopenia has been identified as a predictor of worse OS in advanced sarcomas [23]. In preclinical models of sarcoma, antitumor efficacy of doxorubicin is linked to the presence of an intact innate immune system that can produce interferon-alpha and induce a “type-1 interferon” response in tumor cells [24]. These investigators then performed a retrospective analysis of patients with high risk breast carcinoma receiving adjuvant anthracycline-based therapy. They identified an improved metastasis-free survival for those with tumors harboring a high rather than low expression of this type-1 interferon signature [24]. Further work is needed to explore the molecular and immunologic heterogeneity of euLMS and the impact of these aberrations on clinical outcomes. This will help “personalize” treatment strategies for these patients that will help them live longer with advanced disease.

## 5. Conclusions

In the largest cohort to date to analyze outcomes in extrauterine leiomyosarcoma, factors that influenced prognosis from the time of metastatic disease were sex, age, and metastatic disease burden. No significant difference in clinical activity could be detected between anthracycline-based and gemcitabine-based therapies in the first-line setting. Those whose tumors responded to first-line therapy tended to live longer, suggesting that future studies in this sarcoma subtype should focus on understanding the genetic and tumor microenvironmental factors that underlie this heterogeneity.

## Supplementary Material

Table with detailed listing of first-line systemic therapy regimens and objective responses by specific regimen.

## Figures and Tables

**Table 1:** Patients' demographics, *n* = 215.

Age (range)	56.4 (23.4–85.4)
Tumor size (range)	9 (2–30)
Number of metastatic sites (range)	2 (1–8)
Presentation status	
Primary disease	132 (61)
Metastatic disease	83 (39)
Gender	
Female	135 (63)
Male	80 (37)
Primary tumor size	
<5 cm	34 (16)
5–10 cm	81 (38)
>10 cm	80 (37)
Unknown primary	10 (5)
Known primary, unknown size	10 (5)
KPS	
60	2 (1)
70	8 (4)
80	46 (21)
90	65 (30)
100	22 (10)
Unknown	72 (33)
Primary site	
A/P or RP	151 (70)
Extremity	45 (21)
Trunk	9 (4)
Unknown primary	10 (5)
Margins	
Negative	95 (44)
Positive	45 (21)
Gross disease	3 (1)
N/A—no surgery	47 (22)
Unknown	25 (12)
Grade	
Low	7 (3)
Intermediate	5 (2)
High	176 (82)
Unknown	27 (14)
Depth	
Superficial	5 (2)
Deep	196 (91)
Unknown	14 (7)
Number of metastatic sites	
<3	144 (67)
≥3	71 (33)
Lung metastases	
No	102 (47)
Yes	113 (53)
Liver metastases	
No	159 (74)
Yes	56 (26)

Numbers may not add to 100 due to rounding. KPS: Karnofsky Performance Status; A/P: abdomen/pelvis, RP: retroperitoneal; and N/A: not applicable.

**Table 2:** Univariate analysis of clinicopathologic features on OS from time of metastasis.

Variable	*n*	Number of events	Median OS	*p* value
Time to metastatic disease (years, continuous)	215	190	—	0.036^*∗∗*^
Age				
≤56	104	85	2.79	**0.006**
>56	111	105	2.38	
Primary tumor size				
<5 cm	34	26	2.92	0.711
5–10 cm	81	75	2.54	
>10 cm	80	69	2.69	
Gender				
Female	135	115	2.91	**0.005**
Male	80	75	2.19	
Primary site				
A/P or RP	151	131	2.73	0.097
Extremity	45	40	2.54	
Trunk	9	9	1.74	
Unknown	10	10	2.51	
Margins				
Negative	95	82	2.58	0.801
Positive	48	43	2.9	
Grade				
Low/intermediate	12	11	4.17	0.165
High	176	155	2.54	
Depth				
Superficial	5	4	1.49	0.538
Deep	196	173	2.69	
Number of metastatic sites				
<3	144	125	2.83	**0.002**
≥3	71	65	2.03	
KPS				
≤70	10	9	2.35	0.115
>70	133	116	2.91	
Lung metastases				
No	102	90	2.46	0.586
Yes	113	100	2.71	
Liver metastases				
No	159	143	2.71	0.529
Yes	56	47	2.37	
Treatment for recurrence^*∗*^				
Chemo only	49	40	2.37	0.077
Local therapy only	33	30	3.06	
Chemo + local treatment	40	35	2.98	
Year of metastatic disease				
Before 2000	51	48	2.23	0.387
2000 and after	164	142	2.71	

^*∗*^Treatment for recurrence only analyzed in *n* = 132 patients presenting with primary disease. ^*∗∗*^ *p* value comes from score test. A/P: abdomen/pelvis, RP: retroperitoneal, and chemo: chemotherapy.

**Table 3:** Multivariate analysis of clinicopathologic features on OS from time of metastasis.

	HR	95% lower	95% upper	*p* value
Age (>56 versus ≤56)	1.36	1.01	1.84	0.045
Gender (M versus F)	1.42	1.05	1.92	0.023
Number of metastatic sites (≥3 versus <3)	1.49	1.10	2.03	0.011
Time to metastatic disease (cont.)	0.92	0.84	1.01	0.078

**Table 4:** First-line systemic therapy regimens and median time to treatment failure (TTF).

Systemic therapy	*n*	Median TTF (months)
Gemcitabine + docetaxel	31	5.2
Gemcitabine	12	4.3
Doxorubicin-alkylator combo	20	3.1
Liposomal doxorubicin	12	2.9
Doxorubicin	6	3.3
Alkylator, single	3	1.8
Tyrosine kinase inhibitors	7	9.3
Other combos	13	7.2
Other single agents	9	1.6

**Table 5:** Association between clinicopathologic features associated with OS and first-line systemic therapy regimens. The variability of the number of metastatic sites is presented as the 75th percentile to better reflect the true variability rather than displaying the median.

Systemic therapy	Median age (years)	Female : male ratio	Median time to metastases (years)	Number of metastatic sites, 75th percentile^*∗*^
Gemcitabine + docetaxel	56.5	2.6	0.23	5
Gemcitabine	72	1.3	0.78	2
Doxorubicin-alkylator combo	56.2	1	0	2
Liposomal doxorubicin	66.7	2.9	1.0	3.5
Doxorubicin	58.7	5	0	5
Alkylator, single	65.4	2	0.81	3.5
Tyrosine kinase inhibitors	51.7	1.3	1.1	3.5
Other combos	58.7	1.6	0.56	2
Other single agents	51.8	2.3	1.1	3
None	55.2	1.6	1.5	2
*p* value	0.20	0.66	0.04	0.10

^*∗*^Defined as number at which 75% had fewer and 25% had greater metastases.
