# Supplementary material for: Overall Survival and Response to Systemic Therapy in Metastatic Extrauterine Leiomyosarcoma
Source: Sarcoma. 2016 May 29;2016:3547497. doi: 10.1155/2016/3547497 (PMC4903146; doi:10.1155/2016/3547497)
Supplement: Supplementary file 1 — Table with detailed listing of first-line systemic therapy regimens and objective responses by specific regimen. [file 3547497.f1.docx]

**Supplemental Table 1**.

|  |  | **Best Objective Response** | | | |  |
| --- | --- | --- | --- | --- | --- | --- |
|  | **N** | **CR** | **PR** | **SD** | **PD** | **Total Objective Response**  **(%, 95% CI)** |
| **Gemcitabine + Docetaxel** | **31** | **1** | **7** | **15** | **8** | **8/31 (26%, 12% - 45%)** |
| **Gemcitabine** | **12** | **0** | **3** | **6** | **3** | **3/12 (25%, 5% - 57%)** |
| **Doxorubicin-Alkylator Combo** | **20** | **2** | **3** | **14** | **1** | **5/20 (25%, 9% - 49%)** |
| AIM | 7 | 0 | 2 | 5 | 0 |  |
| MAID | 3 | 1 | 0 | 2 | 0 |  |
| dox + dacarbazine | 10 | 1 | 1 | 7 | 1 |  |
| **Liposomal Doxorubicin** | **12** | **0** | **0** | **7** | **5** | **0/12 (0%)** |
| **Doxorubicin** | **6** | **0** | **0** | **4** | **2** | **0/6 (0%)** |
| **Alkylator Single** | **3** | **0** | **0** | **0** | **3** | **0/3 (0%)** |
| temozolomide | 2 | 0 | 0 | 0 | 2 |  |
| dacarbazine | 1 | 0 | 0 | 0 | 1 |  |
| **Tyrosine Kinase Inhibitors** | **7** | **0** | **0** | **7** | **0** | **0/7 (0%)** |
| sorafenib | 6 | 0 | 0 | 6 | 0 |  |
| sunitinib | 1 | 0 | 0 | 1 | 0 |  |
| **Other combo** | **13** | **0** | **4** | **8** | **1** | **4/13 (31%, 9% - 61%)** |
| gem + doce + bevacizumab | 4 | 0 | 3 | 1 | 0 |  |
| sorafenib + dacarbazine | 4 | 0 | 1 | 3 | 0 |  |
| dox + experimental agents | 2 | 0 | 0 | 2 | 0 |  |
| liposomal dox + dacarbazine | 2 | 0 | 0 | 2 | 0 |  |
| 5-FU + experimental agent | 1 | 0 | 0 | 0 | 1 |  |
| **Other Single Agents** | **9** | **0** | **1** | **2** | **6** | **1/9 (11%, 0.3% - 48%)** |
| bortezomib | 2 | 0 | 1 | 0 | 1 |  |
| trabectedin | 1 | 0 | 0 | 0 | 1 |  |
| other experimental agents | 6 | 0 | 0 | 2 | 4 |  |
| **Total** | **113** | **3** | **18** | **63** | **29** | **21/113 (19%, 12% - 27%)** |

**Supplemental Table 1**. First-line systemic therapy regimens and objective responses. CR = complete response, PR = partial response, SD = stable disease, PD = progressive disease; AIM = doxorubicin, ifosfamide, mesna; MAID = mesna, doxorubicin, ifosfamide, dacarbazine; Dox = doxorubicin; gem = gemcitabine; doce = docetaxel.
